# Household Poverty and Obesity in Children With Acute Lymphoblastic Leukemia: A Report From COG-AALL03N1

**DOI:** 10.1002/mpo.70008

**Published:** 2025-11-19

**Authors:** Karnika Mehrotra, Yanjun Chen, Joshua Richman, Lindsey Hageman, Wendy Landier, Smita Bhatia, Aman Wadhwa

**Affiliations:** 1 University of Alabama at Birmingham Heersink School of Medicine, Birmingham, Alabama, USA; 2 Institute for Cancer Outcomes and Survivorship, University of Alabama at Birmingham, Birmingham, Alabama, USA; 3 Department of Surgery, University of Alabama at Birmingham, Birmingham, Alabama, USA; 4 Division of Pediatric Hematology/Oncology, University of Alabama at Birmingham, Birmingham, Alabama, USA

**Keywords:** acute lymphoblastic leukemia, obesity, poverty

## Abstract

Whether children with acute lymphoblastic leukemia (ALL) living in household poverty are at an increased risk of obesity remains unknown. We address this gap using data from Children’s Oncology Group study AALL03N1. Study participants reported annual household income, which was used to categorize poverty from year-specific US Census Bureau thresholds. Obesity was determined using height and weight at study enrollment. Multivariable logistic regression demonstrated 1.5-fold greater odds of obesity (95% confidence interval = 1.0–2.3, *p* = 0.04) among children with ALL living in poverty. These findings identify a vulnerable group at risk of obesity.

## 1 | Introduction

Children with obesity and acute lymphoblastic leukemia (ALL) experience greater treatment-related toxicity [1] as well as relapse [1, 2], underscoring the need to improve our understanding of factors associated with the risk of obesity. In the United States, the prevalence of obesity among children and adolescents has increased over the past decades [3], in part due to exposure to adverse social determinants of health [4]. Household poverty (through its negative effects on nutrition, physical activity, and sleep) is associated with an increased risk of childhood obesity in the general population [4, 5]. Among children with ALL, factors associated with obesity during treatment include younger age at diagnosis [6], female sex [6, 7], exposure to cranial irradiation ≥ 20 Gy [8], higher body mass index (BMI) at diagnosis [6], corticosteroid exposure [9], and weight gain during induction therapy [9]. Whether household poverty is associated with obesity during treatment in children with ALL remains unknown. We address this gap by using data from Children’s Oncology Group study AALL03N1.

## 2 | Methods and Results

AALL03N1was an observational study (primary aim: adherence to 6-mercaptopurine during maintenance therapy for ALL) that enrolled patients ≤ 21 years (y) at ALL diagnosis who had entered maintenance in first remission after completing ≥ 6 months of maintenance therapy and had ≥ 6 months remaining. COG participating sites had approval from institutional review boards; patients (if ≥ 18 y) and/or parents/legal guardians (if < 18 y) provided written informed consent prior to enrollment. Participants self-reported race/ethnicity, annual household income, and household structure (single-parent household vs. other). Age at ALL diagnosis and study enrollment, sex, National Cancer Institute risk grouping, ALL subtype, blast cytogenetics, therapeutic protocol, and height and weight at monthly visits were provided by participating sites.

Participants reported annual household income at study enrollment in the following categories: < $20,000; $20,000–$49,999; $50,000–$74,999; $75,000–$999,999, and ≥ $100,000. We used the midpoint of these ranges as the annual household income (e.g., $35,000 if a parent reported income as $20,000–$49,999). We used year-specific federal poverty threshold data from the US Census Bureau, accounting for the number of household members. We categorized patients with annual household income below the year-specific poverty thresholds as living in household poverty (primary exposure) [10]. The primary outcome was obesity at study enrollment. Height (m) and weight (kg) at study enrollment were used to calculate BMI ( = weight/[height]^2^). For patients ≤ 19y, BMI was operationalized as percentiles using US Center for Disease Control and Prevention (CDC) data. Obesity was defined as a BMI percentile ≥ 95 for patients ≤ 19 y, or an absolute BMI ≥ 30 kg/m^2^ for patients > 19 y per CDC guidelines [11]. Median BMI percentiles throughout the study period were also calculated.

Demographic and clinical characteristics by household poverty were summarized. We used logistic regression to examine the association between household poverty and obesity at enrollment. A multivariable model adjusted for variables with *p* < 0.1 on univariate models retained age at enrollment, sex, race/ethnicity, and therapeutic protocol in the final model regardless of significance, given known associations with obesity. Generalized estimating equations were used to examine the association between household poverty and change in BMI percentile, adjusting for covariates. Statistical analyses used SPSS and SAS; two-sided *p* values < 0.05 were considered significant.

Of the 742 participants enrolled in AALL03N1, 134 (18.1%) were excluded due to missing data needed to calculate poverty (*n* = 110) or height or weight (*n* = 24), yielding 608 patients. Excluded patients were older at study enrollment (median, 8 y [range, 2–21] vs. 6 y [2–19], *p* = 0.001), more likely to report race/ethnicity as Asian (21.6% vs. 13.5%) or non-Hispanic White (38.8% vs. 31.1%), and less likely to report race/ethnicity as Hispanic (23.9% vs. 36.5%, *p* = 0.005) or live in a single-parent household (7.7% vs. 15.6%, *p* = 0.03).

Table 1 highlights demographic characteristics of the cohort. Among those living in household poverty, there was an over-representation of African American (24.7% vs. 16.7%, *p* < 0.001) and Hispanic (60.5% vs. 27.2%, *p* < 0.001) patients as well as patients living in single-parent household (32.5% vs. 9.1%, *p* < 0.001). Overall, 184 (30.3%) participants had obesity at study enrollment. Multivariable analysis showed that patients living in household poverty were at 1.5-fold greater odds of obesity at study enrollment compared to those not living in household poverty (95% confidence interval = 1.0–2.3, *p* = 0.04), after adjusting for age at study enrollment, sex, race/ethnicity, and therapeutic protocol (Table 2). Figure S1 highlights the median BMI percentile over the study period by household poverty. Household poverty at study enrollment was not associated with BMI percentile change during the study period (*β*-coefficient = −1.4, standard error = 0.9, *p* = 0.1).

## 3 | Discussion

Poverty is associated with an increased risk of obesity in children without cancer [12]; however, this association among children with ALL has remained unexamined. Existing literature in children with ALL has largely identified demographic factors and therapeutic exposures as risk factors for obesity. We identify the impact of household poverty on obesity. Given the association of obesity with inferior outcomes among children with ALL [1, 2], recognition of intervenable risk factors is critical to allow for the development of targeted interventions. While ongoing studies are investigating the effect of caloric and nutrient restriction on reducing weight gain during induction therapy with promising early results [13], known risk factors for obesity in children with ALL are essentially non-modifiable [6–8]. COG protocols have reduced the frequency of corticosteroid pulses during maintenance; whether this will lead to lower weight gain during maintenance remains unknown. Thus, identification of household poverty as a risk factor for obesity presents an additional avenue for testing targeted interventions. We did not, however, identify household poverty as a risk factor for differential change in BMI. Longer follow-up studies are needed to further understand the effect of household poverty on changes in BMI.

We propose several mechanisms to explain our observation. First, in the general population, obesity is more prevalent among children living in an adverse built environment [14], exposed to inferior infrastructure, poor sleep, and paucity of safe spaces to exercise and play [4, 5, 15]. Existing data have demonstrated increased obesity among cancer survivors living in neighborhoods with lower walkability [16]. Second, living in food deserts may limit access to nutritious foods, requiring families to rely on high-calorie food, leading to unhealthy consumption of macronutrients [17]. Pediatricians screen for food insecurity using resources offered by the American Academy of Pediatrics. However, pediatric oncologists assume responsibility for all aspects of care during treatment of cancer and thus need to screen and address food insecurity among children with cancer. Though the mechanisms of poverty contributing to obesity are likely multifactorial, leveraging existing interventions could reduce the risk of obesity among children with ALL. The Supplemental Nutrition Assistance Program can reduce the barrier of high cost for healthy food, leading to improved nutrition, although recent changes in eligibility could limit its effectiveness in attenuating cost-related barriers to nutritious foods for children living in poverty [18]. Programs targeting nutrition for pediatric cancer survivors have demonstrated feasibility and lowered food insecurity [19]. Finally, behavioral treatment strategies have also been shown to be beneficial in reducing obesity among adults [20].

Our study has limitations. AALL03N1 did not collect anthropometric data at ALL diagnosis, precluding the ability to adjust for BMI at diagnosis. While AALL03N1 captured therapeutic protocols, data were missing on corticosteroid type and cumulative dose. However, we tested the association while adjusting for therapeutic protocol as a surrogate.

In conclusion, our findings highlight the need for specialized interventions for children with ALL living in poverty to mitigate the risk of adverse outcomes associated with obesity in this population.

## Supplementary Material

Supplemental Figure **Supplemental Figure 1:** Median body mass index (BMI) percentile across study months.

Additional supporting information can be found online in the Supporting Information section.

## Figures and Tables

**TABLE 1 |:** Demographic and disease characteristics of the cohort by household poverty.

Variable	Entire cohort (*n* = 608)	No household poverty (*n* = 438)	Household poverty (*n* = 170)	*p*
Age at study enrollment in years				
Median (range)	6.0 (2–19)	6.0 (2–19)	6.0 (2–19)	0.6
Mean (± SD^1^)	7.6 (4.4)	7.6 (4.4)	7.6 (4.5)	0.9
Sex, *n* (%)				
Male	417 (68.6)	293 (66.9)	124 (72.9)	0.1
Race/ethnicity, *n* (%)				
African American or Black	115 (18.9)	73 (16.7)	42 (24.7)	< 0.001
Asian	82 (13.5)	68 (15.5)	14 (8.2)	
Hispanic	222 (36.5)	119 (27.2)	103 (60.5)	
Non-Hispanic White	189 (31.1)	178 (40.6)	11 (6.5)	
Household structure, *n* (%)				
Single-parent household	94 (15.6)	40 (9.1)	54 (32.5)	< 0.001
ALL^2^ subtype, *n* (%)^a^				
B-lymphoblastic leukemia	539 (89.4)	338 (89.4)	151 (89.3)	0.9
T-lymphoblastic leukemia	64 (10.6)	46 (10.6)	18 (10.7)	
NCI^3^ risk group, *n* (%)				
Standard risk	357 (58.7)	260 (59.5)	97 (57.7)	0.7
Cytogenetics, *n* (%)^b^				
Favorable	240 (41.9)	183 (43.9)	57 (36.5)	0.03
Neutral	303 (52.9)	218 (52.3)	85 (54.5)	
Unfavorable	30 (5.2)	16 (3.8)	14 (9.0)	
Therapeutic protocol, *n* (%)				
AALL0331	181 (29.8)	127 (29.0)	54(31.8)	0.2
CCG-1991	139 (22.9)	108 (24.7)	31 (18.2)	
AALL0232	122 (20.1)	80 (18.3)	42 (27.4)	
CCG-1961	76 (12.5)	55 (12.6)	21 (12.4)	
Other	90 (14.8)	68 (15.5)	22 (12.9)	

*Note: p* value compares patients living in household poverty versus those not living in household poverty.

Abbreviations: ALL, acute lymphoblastic leukemia; NCI, National Cancer Institute; SD, standard deviation.

a Data were missing for ALL subtype (*n* = 5).

b Data were missing for cytogenetics (*n* = 35).

**TABLE 2 |:** Association between household poverty and obesity at study enrollment.

Variable	Univariate		Multivariable	
	OR (95% CI)	*p*	OR (95% CI)	*p*
Poverty (reference: no household poverty)				
Household poverty	1.8 (1.2–2.6)	0.002	1.5 (1.0–2.3)	0.04
Age at study enrollment				
Per year increase	1.0 (0.9–1.0)	0.9	1.0 (0.9–1.0)	0.9
Sex (ref: female)				
Male	1.2 (0.8–1.6)	0.5	0.9 (0.6–1.3)	0.5
Race/ethnicity (ref: non-Hispanic white)				
African American or Black	1.7 (1.1–2.6)	0.01	1.3 (0.8–2.2)	0.3
Asian	0.6 (0.3–1.1)	0.1	0.5 (0.3–1.0)	0.06
Hispanic	1.4 (0.9–2.3)	0.2	1.2 (0.7–2.1)	0.5
Household structure (ref: nuclear family)				
Single parent	0.8 (0.5–1.3)	0.4	—	—
ALL subtype (ref: B-ALL)				
T-ALL	1.2 (0.7–2.2)	0.5	—	—
NCI risk group (ref: standard risk)				
High risk	1.2 (0.8–1.7)	0.4	—	—
Blast cytogenetics (ref: neutral)				
Favorable	1.1 (0.8–1.7)	0.5	—	—
Unfavorable	1.3 (0.6–2.9)	0.5	—	—
Therapeutic protocol (ref: AALL0331)				
CCG-1991	1.0 (0.6–1.6)	0.9	1.0 (0.6–1.8)	0.9
AALL0232	1.5 (0.9–2.5)	0.09	1.6 (0.9–2.8)	0.1
CCG-1961	0.9 (0.5–1.6)	0.7	0.9 (0.5–1.8)	0.8
Other	0.7 (0.4–1.2)	0.2	0.8 (0.4–1.4)	0.4

Abbreviations: 95% CI, 95% confidence interval; ALL, acute lymphoblastic leukemia; NCI, National Cancer Institute; OR, odds ratio.

## Data Availability

Data from COG-AALL03N1 are available upon reasonable request from Smita Bhatia (smitabhatia@uabmc.edu).

## Abbreviations:

ALL: acute lymphoblastic leukemia
BMI: body mass index
CDC: Centers for Disease Control and Prevention
SDOH: social determinants of health
Y: years
