# Supplementary figures and images for: Household Poverty and Obesity in Children With Acute Lymphoblastic Leukemia: A Report From COG-AALL03N1
Source: Pediatr Blood Cancer. Author manuscript; Available in PMC 2026 Mar 30. (PMC13034799; doi:10.1002/mpo.70008)

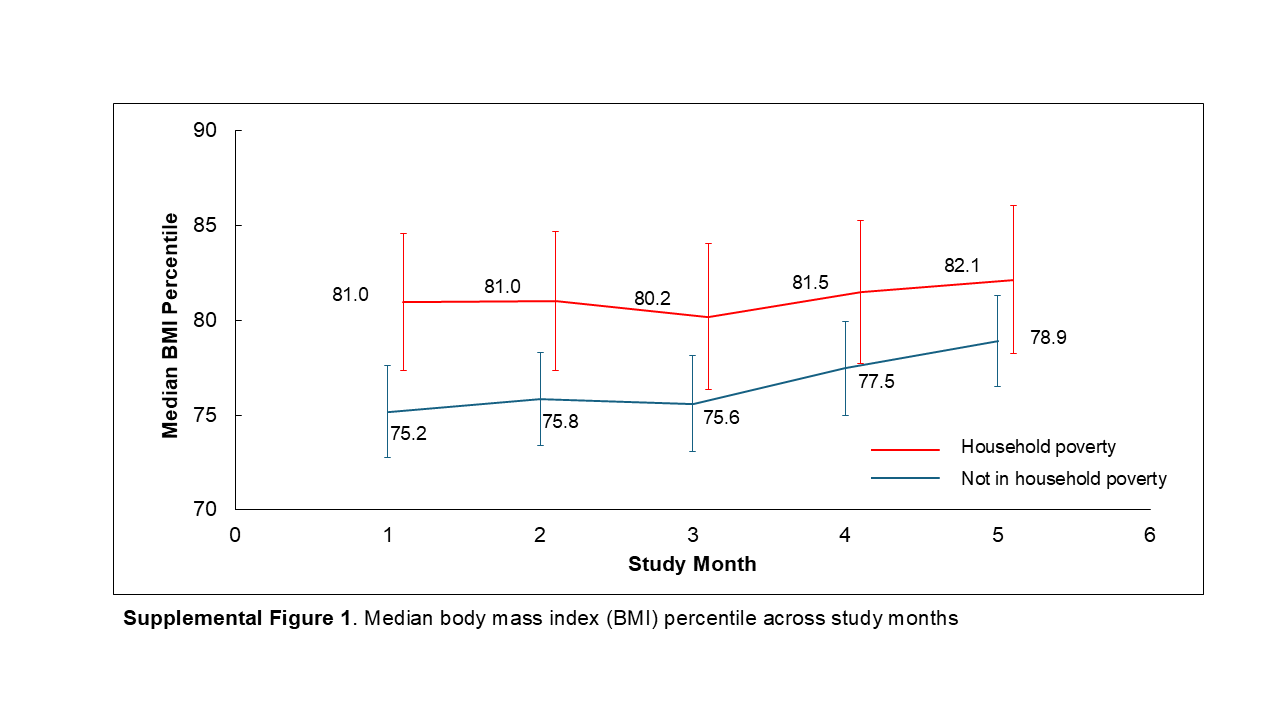

Supplement: Supplemental Figure — 1: Median body mass index (BMI) percentile across study months. [file NIHMS2155131-supplement-Supplemental_Figure.tif]
